# Radiologist Involvement in Radiation Oncology Peer Review: A Systematic Review and Meta-Analysis

**DOI:** 10.1001/jamanetworkopen.2024.52667

**Published:** 2024-12-27

**Authors:** Ryan T. Hughes, Niema B. Razavian, Sydney Smith, Ralph B. D’Agostino, Paul M. Bunch, Janardhana Ponnatapura, Trevor J. Royce, James D. Ververs, Chandylen L. Nightingale, Kathryn E. Weaver, Michael K. Farris

**Affiliations:** 1 Department of Radiation Oncology, Wake Forest University School of Medicine, Winston-Salem, North Carolina; 2 Department of Biostatistics and Data Science, Wake Forest University School of Medicine, Winston-Salem, North Carolina; 3 Department of Radiology, Wake Forest University School of Medicine, Winston-Salem, North Carolina; 4 Department of Social Sciences and Health Policy, Wake Forest University School of Medicine, Winston-Salem, North Carolina

## Abstract

**Importance:**

Radiotherapy (RT) plan quality is an established predictive factor associated with cancer recurrence and survival outcomes. The addition of radiologists to the peer review (PR) process may increase RT plan quality.

**Objective:**

To determine the rate of changes to the RT plan with and without radiology involvement in PR of radiation targets.

**Data Sources:**

PubMed, Scopus, and Web of Science were queried for peer-reviewed articles published from inception up to March 6, 2024. Search terms included key words associated with PR of contoured targets for the purposes of RT planning with or without radiology involvement.

**Study Selection:**

Studies reporting PR of contoured radiation targets with or without radiology involvement. Studies were excluded if they lacked full text, reported clinical trial–specific quality assurance, or reported PR without dedicated review of RT targets.

**Data Extraction and Synthesis:**

Data were extracted according to Preferred Reporting Items for Systematic Reviews and Meta-Analyses guidelines. Titles and abstracts were screened by 2 reviewers. In the case of discordance, discussion was used to reach consensus regarding inclusion for full-text review. RT plan changes were characterized as major when the change was expected to have a substantial clinical impact, as defined by the original study. Pooled outcomes were estimated using random-effects models.

**Main Outcomes and Measures:**

Primary outcome was pooled rate of RT plan changes. Secondary outcomes included pooled rates of major and minor changes to RT targets or organs at risk.

**Results:**

Of 4185 screened studies, 31 reporting 39 509 RT plans were included (390 with radiology and 39 119 without). The pooled rate of plan changes was 29.0% (95% CI, 20.7%-37.2%). Radiologist participation in PR was associated with significant increases in plan change rates (49.4% [95% CI, 28.6%-70.1%] vs 25.0% [95% CI, 17.0%-33.1%]; *P* = .02) and in clinically relevant major changes (47.0% [95% CI, 34.1%-59.8%] vs 10.2% [95% CI, 4.6%-15.8%]; *P* < .001). There was no difference in minor changes (15.2% [95% CI, 9.7%-20.6%] vs 13.8% [95% CI, 9.3%-18.3%]; *P* = .74). Subgroup analyses identified increases in the rates of changes to the gross tumor and planning target volumes with radiology-based PR. The highest rates of plan changes were observed in head and neck or lung cancer studies, studies performing PR prior to RT planning, and prospective studies.

**Conclusions and Relevance:**

In this systematic review and meta-analysis of radiation oncology PR of contoured targets, radiologist involvement in peer review was associated with a significant increase in the rate of total and clinically meaningful changes to the RT targets with no change in minor change rates. These results support the value of interdisciplinary collaboration with radiology during RT planning.

## Key Points

**Question:**

What is the association of radiologist involvement in radiation oncology peer review with the radiotherapy plan?

**Findings:**

In this systematic review and meta-analysis of 31 studies reporting 39 509 cases, significantly higher pooled rates of radiotherapy plan changes were observed with (49.4%) vs without (25.0%) radiologist involvement in peer review of contoured targets. Radiologist involvement was also associated with increases in major (47.0% vs 10.2%) but not minor (15.2% vs 13.8%) changes.

**Meaning:**

These results support the value of interdisciplinary collaboration with radiology during radiotherapy planning.

## Teaser

This systemic review and meta-analysis assess published studies on radiation contour peer review to better understand the potential association of radiologist input with radiotherapy treatment plans for patients with cancer.

## Introduction

Peer review (PR) of radiotherapy (RT) treatment plans is critical for radiation oncologists to ensure high-quality and safe treatment delivery.^1^ This process is a requirement for site accreditation by radiation oncology governing bodies such as the American College of Radiology and the American Society for Radiation Oncology. Conventional PR consists of standing meetings in which a patient’s clinical scenario is presented and a general overview of their RT plan, including the site to be treated and RT prescription, is provided. While the specifics of the PR workflow vary by institution, the time spent reviewing each case is brief (approximately 2-3 minutes), and not all cases are reviewed.^2,3,4^ Given this format, it is challenging to ensure that all sites of disease are appropriately delineated and that normal tissues are adequately protected. To highlight this problem, a prospective study^5^ inserted erroneous plans into routine radiation oncology chart rounds and found that only 55% of errors were detected. Moreover, timeliness of PR is also critical: once the RT plan is finalized, the time and effort required to modify the plan based on reviewer feedback presents a barrier to consistent implementation of PR recommendations.

To address these challenges, multiple institutions have transitioned to focused PR of contoured targets and normal tissue structures.^3,6,7,8,9,10,11^ This process is critically important to radiation oncologists, since target and normal tissue structures are unique to each clinical scenario, and their delineation is a leading source of interphysician variability.^12^ Among modern RT techniques, one common source of variability is image interpretation: to develop highly conformal RT plans, radiation oncologists have become increasingly dependent on their ability to interpret multiple imaging modalities (eg, magnetic resonance imaging, positron emission tomography, computed tomography) during the RT planning process. While radiation oncologists are classically trained to interpret clinical scenarios and understand distribution of gross disease and potential routes of spread, radiation oncology residents are provided with limited formal training in diagnostic image interpretation. Despite 87% of residents agreeing that a strong radiological knowledge base is moderately to extremely important, more than 60% feel only “somewhat confident” in radiology at graduation.^13,14,15,16,17,18^ In radiation oncology residency, radiological instruction is primarily in the form of multidisciplinary tumor board imaging review and on-the-job training during routine clinical care.^12^ Guidelines for RT target delineation are available, but these only provide a framework and do not improve image interpretation skills.^19,20^ As a result, significant variability in tumor delineation may exist between radiation oncologists and radiologists.^21^

To address these challenges and bridge gaps in multidisciplinary care, some institutions have developed PR practices that embed radiologists into PR conferences to provide a more detailed radiological review of proposed treatments.^13,14,15,16,17,18^ We performed a systematic review and meta-analysis of published studies on radiation contour PR to better understand the association of radiologist input with changes to RT treatment plans.

## Methods

We performed a systematic review of the literature and meta-analysis according to the Preferred Reporting Items for Systematic Reviews and Meta-analyses (PRISMA) reporting guideline.^22^ This study was prospectively registered in the PROSPERO database (CRD42024544451). Three databases (PubMed, Web of Science, and Scopus) were queried using standardized search terms to identify studies published from inception to March 6, 2024, that reported PR of contoured targets for the purposes of RT planning with or without radiology involvement. Search terms included (*quality assurance* OR *peer review* OR *chart rounds*) AND (*contour* OR *contours* OR *contouring* OR *segmented* OR *delineated* OR *delineation* OR *target* OR *targets* OR *volume* OR *volume delineation*) AND (*radiotherapy* OR *radiation* OR *radiation therapy*). Titles and abstracts were screened by 2 reviewers (R.T.H. and M.K.F.). In the case of discordance, discussion was used to reach consensus regarding inclusion for full-text review. Details on inclusion and exclusion criteria and the systematic review procedures are described in the eMethods in Supplement 1. After full-text review, factors extracted from the studies included cancer diagnosis of patients reviewed, PR timing (before RT planning vs after plan completion), specific structures reviewed in PR, radiologist inclusion, definition of major and minor changes (if applicable), RT plan changes and recommendations for change, and changes to each specific type of target (eg, gross tumor volume [GTV], clinical target volume, planning target volume [PTV]). Quality of studies was assessed using the methodological index for nonrandomized studies.^23^ Scores range from 0 to 24, with higher scores indicating higher study quality; the ideal score is 16 for noncomparative studies and 24 for comparative studies.

The primary outcome of this study was the pooled rate of RT plan change of any kind after PR. These changes could relate to the contoured targets, organs at risk (OARs), prescribed dose, fractionation schedule, RT technique and modality, immobilization, motion management, imaging guidance, bolus or shielding, treatment intent, or treatment cancellation. To adequately capture PR outcomes, a combined outcome measure was used in this analysis: specifically, changes and recommended changes to RT plans were pooled together, regardless of whether they were documented as implemented by the included study. As secondary outcomes, major and minor changes were defined by the individual studies (eTable 1 in Supplement 1); these study-level definitions were then used to categorize major and minor changes for the purposes of this analysis. Generally, major changes were defined as those expected to be clinically meaningful, by preventing geographic miss of gross tumor, affecting disease control outcomes, or affecting relevant toxic effects; minor changes were not expected to have substantial clinical importance. The total number of plan changes (of any type or significance) was extracted from studies, where reported, or calculated as the sum of major and minor changes, when these 2 change levels were reported separately. We evaluated the pooled rate of RT plan changes with and without radiology involvement in PR, as well as the pooled rate of change stratified by major and minor changes. We further looked at the rate of changes with and without radiology involvement specific to GTV, PTV, and OARs. Sensitivity analyses were also performed to assess the association of primary disease site, PR timing (before vs after the dosimetric planning process—the act of creating and finalizing the RT plan based on the contoured target volumes), PR frequency (daily, multiple times per week, weekly, or as needed), and study type (prospective or retrospective) with RT plan changes.

### Statistical Analysis

The primary and secondary outcomes were estimated using a random-effects model, and heterogeneity was assessed using Cochran Q tests and the Higgins *I*^2^ statistic. In cases of high heterogeneity (*I*^2^ > 50%), Cook distance was used to identify outliers, and pooled rates were recalculated after exclusion of outliers. Differences between subgroups were assessed using the omnibus test of moderators (Qm), testing the null hypothesis that all predictive factors entered into the model are unrelated to the observed effect sizes. Because only 1 study^18^ reported the outcomes of 2 groups undergoing PR with and without radiologist participation, models comparing effects of radiologist involvement across studies were not performed. Publication bias was assessed using funnel plots and the Egger regression test for plot symmetry.^24^ Statistical significance was defined using a 2-sided α of .05. All analyses were performed using the metafor package in R, version 4.4.1 (R Project for Statistical Computing),^25^ and RStudio, version 2024.09.0 + 375.^26^

## Results

### Characteristics of Included Studies

Systematic review identified 4185 studies for screening; 62 underwent full text review, and 31 with 39 509 cases of PR were included in the pooled analysis (eFigure 1 in Supplement 1).^3,6,7,8,9,10,11,13,14,15,16,17,18,27,28,29,30,31,32,33,34,35,36,37,38,39,40,41,42,43,44^ Characteristics of the included studies are presented in eTable 1 in Supplement 1. Studies were published between 2014 and 2024; 18 were prospective^3,6,10,11,13,15,17,18,27,29,33,34,37,38,39,40,41,44^ and 13 were retrospective.^7,8,9,14,16,28,30,31,32,35,36,42,43^ By specific cancer diagnosis, PR of head and neck cancer (n = 11)^13,14,15,17,18,27,30,36,37,38,40^ and multiple diagnoses (n = 12)^3,6,7,8,9,10,11,16,31,33,42,43^ were most common, followed by stereotactic body RT (SBRT) at various sites (n = 3),^32,35,39^ and lung cancer (n = 2)^29,44^; the remaining 3 studies included patients with other diagnoses including breast,^34^ gynecologic,^41^ or hematologic cancers.^28^ Fifteen studies^13,14,15,16,17,18,27,28,32,36,37,38,40,41,44^ reported PR prior to initiation of treatment planning, 9 studies^3,7,9,29,31,34,35,42,43^ reviewed contours after planning, and 7 studies^6,8,10,11,30,33,38^ reported a mix of preplanning and postplanning PR. Twenty-four studies^3,7,8,9,10,11,13,14,15,16,17,18,27,30,31,33,34,35,36,37,38,40,41,42,43^ indicated the clinical importance of PR changes, while 7 studies^6,16,17,28,29,32,39^ did not. Clinical importance was reported as major vs minor changes (most common), clinically significant vs clinically insignificant (or noncritical), letter grades (ie, A [no changes], B [minor changes], and C [major changes]), or stoplight scoring (red indicating major changes and yellow indicating minor changes). The definitions of the severity of major and minor changes are shown in eTable 1 in Supplement 1. In terms of contours examined during PR, 30 studies reported reviewing target structures: 15 studies^3,9,10,18,27,29,31,33,34,35,39,40^ reviewed all targets and OARs, 13 studies^8,11,13,14,15,16,17,28,30,32,36,38,41^ reviewed only targets, and 2 studies^6,37^ reviewed only specific targets and OARs. Regarding the intent of RT for cases undergoing PR, pooled rate of curative intent was 93.7%; palliative intent, 6.3%; definitive intent, 62.0%; and adjuvant or neoadjuvant intent, 35.8% (eTable 2 in Supplement 1). Median study quality (per the methodological index for nonrandomized studies) was 8 (range, 5-10) (eTable 3 in Supplement 1). No evidence of publication bias was identified across studies (Egger test *P* = .56) (eFigure 2 in Supplement 1).

### RT Plan Changes With and Without Radiology Involvement in PR

**Figure 1.**
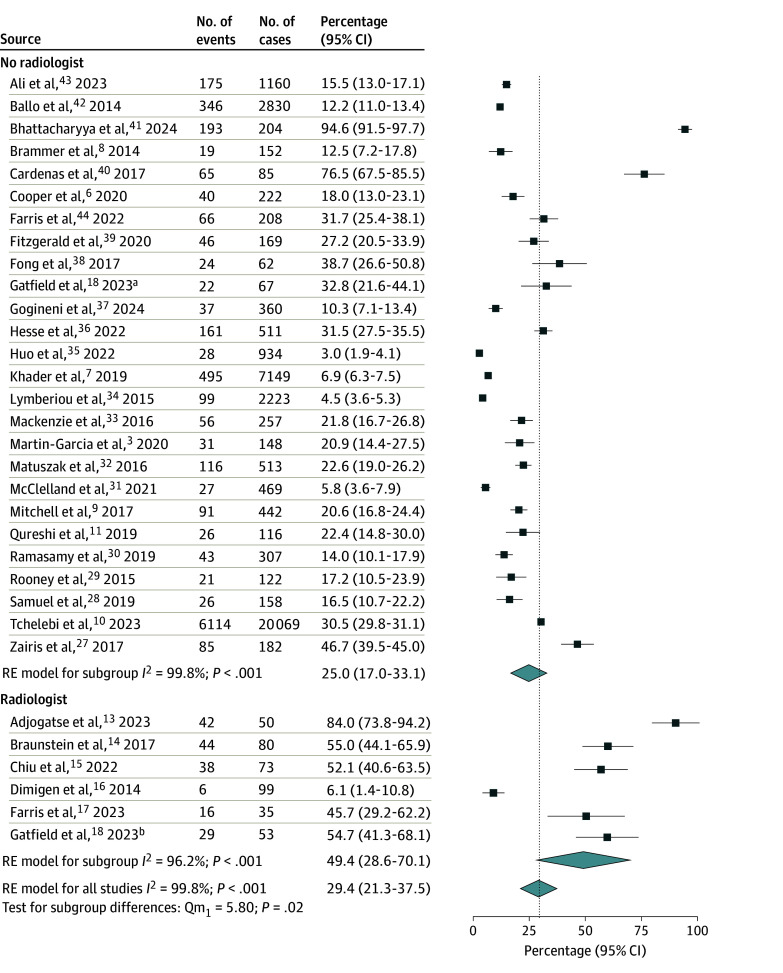
Forest Plot and Pooled Rates of Any Plan Changes by Radiology Involvement in Radiotherapy Peer Review Vertical line indicates the pooled rate for all studies. Light blue diamonds represent pooled rates and 95% CIs. RE indicates random effects. ^a^Subgroup reviewed without radiologist. ^b^Subgroup reviewed with radiologist.

**Figure 2.**
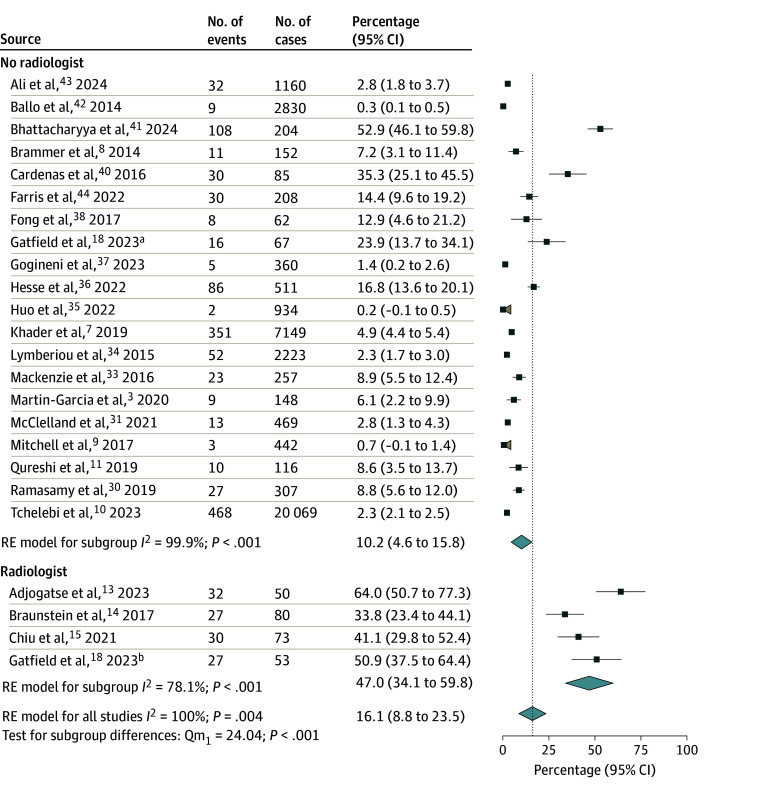
Forest Plot and Pooled Rates of Major Changes to the Radiotherapy Plan by Radiology Involvement Studies that did not differentiate changes as major vs minor (n = 7) were excluded from this analysis. Vertical line indicates the pooled rate for all studies. Tan shaded areas indicate that the lower confidence bound crosses 0; light blue diamonds represent pooled rates and 95% CIs. RE indicates random effects. ^a^Subgroup reviewed without radiologist. ^b^Subgroup reviewed with radiologist.

**Figure 3.**
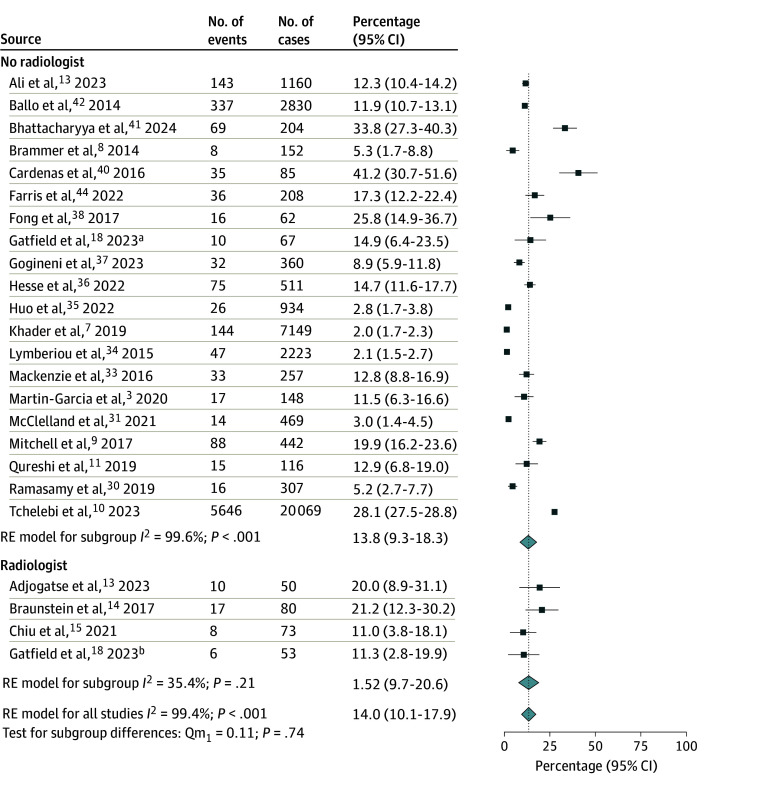
Forest Plot and Pooled Rates of Minor Changes to the Radiotherapy Plan by Radiology Involvement Studies that did not differentiate changes as major vs minor (n = 7) were excluded from this analysis. Vertical line indicates the pooled rate for all studies. Light blue diamonds represent pooled rates and 95% CIs. RE indicates random effects. ^a^Subgroup reviewed without radiologist. ^b^Subgroup reviewed with radiologist.

A total of 6 studies (390 cases)^13,14,15,16,17,18^ reported outcomes of PR in the presence of radiologists, while 25 studies (39 1196 cases)^3,6,7,8,9,10,11,27,28,29,30,31,32,33,34,35,36,37,38,39,40,41,42,43,44^ reported PR without radiologist involvement. The pooled rate of RT plan changes was significantly higher among studies that incorporated radiologists in PR than studies that did not at 49.4% (95% CI, 28.6%-70.1%) vs 25.0% (95% CI, 17.0%-33.1%), respectively (*P* = .02) (Figure 1). In the 24 studies that reported major and minor changes,^3,7,8,9,11,13,14,15,17,18,27,30,31,33,34,35,36,37,38,40,41,42,43^ the involvement of radiology in PR was associated with an increased rate of major RT plan changes (47.0% [95% CI, 34.1%-59.8%] vs 10.2% [95% CI, 4.6%-15.8%]; *P* < .001) (Figure 2) but no difference in minor changes (15.2% [95% CI, 9.7%-20.6%] vs 13.8% [95% CI, 9.3%-18.3%]; *P* = .74) (Figure 3). Given the high level of heterogeneity observed, these primary analyses were repeated after the removal of outliers. After removal of outlier studies, heterogeneity remained high in the group that did not include a radiologist, but the outcomes were not substantially different from those observed in the analysis of the total cohort for all changes, major changes, and minor changes.

**Figure 4.**
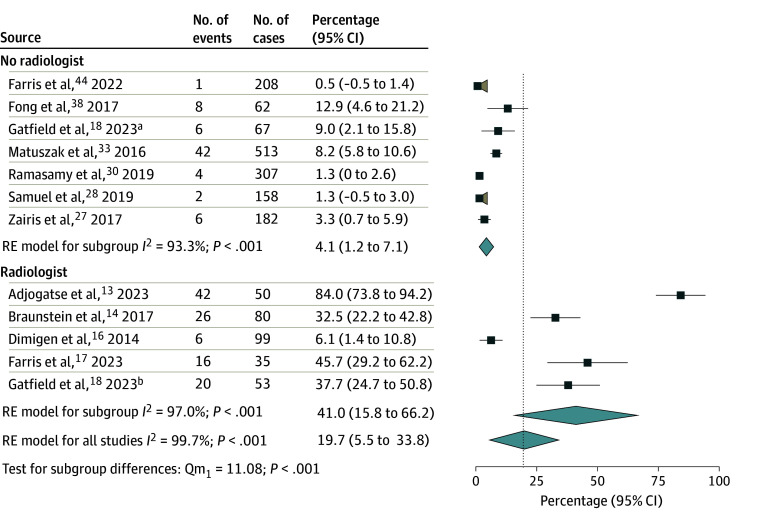
Forest Plot and Pooled Rates of Changes to the Gross Tumor Volume by Radiology Involvement Vertical lines indicate the pooled rates for each subgroup. Tan shaded areas indicate that the lower confidence bound crosses 0; light blue diamonds represent pooled rates and 95% CIs. RE indicates random effects. ^a^Subgroup reviewed without radiologist. ^b^Subgroup reviewed with radiologist.

**Figure 5.**
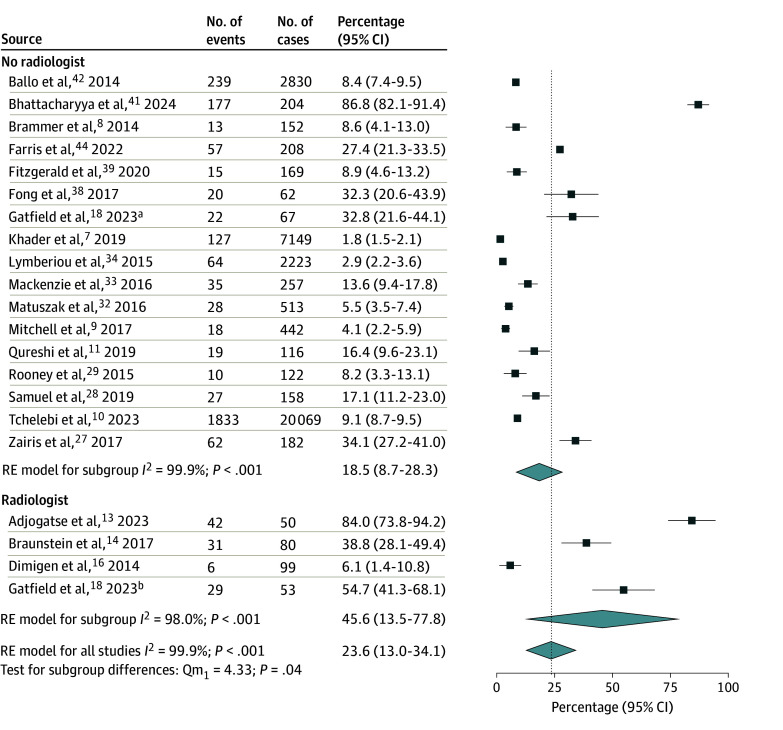
Forest Plot and Pooled Rates of Changes to the Planning Target Volume or Treatment Volume by Radiology Involvement Vertical line indicates the pooled rate for all studies. Light blue diamonds represent pooled rates and 95% CIs. RE indicates random effects. ^a^Subgroup reviewed without radiologist. ^b^Subgroup reviewed with radiologist.

Subgroup analyses were performed on the studies reporting changes to specific contoured volumes because of PR, including GTV (12 studies; 1814 cases),^13,14,16,17,18,27,28,30,32,38,44^ PTV and treatment volumes (21 studies; 35 205 cases),^7,8,9,10,11,13,14,16,17,18,27,28,29,32,33,34,38,39,41,42^ and OARs (12 studies; 31 023 cases).^7,9,10,16,17,18,27,28,34,39,41^ Radiologist-involved PR was associated with significant increases in rates of change to the GTV (41.0% [95% CI, 15.8%-66.2%] vs 4.1% [95% CI, 1.,2%-7.1%]; *P* < .001) (Figure 4) and PTV and target volumes (45.6% [95% CI, 13.5%-77.8%] vs 18.5% [95% CI, 8.7%-28.3%]; *P* = .04) (Figure 5), but not to OARs (4.1% [95% CI, −4.5% to 12.7%] vs 4.3% [95% CI, −0.6% to 9.2%]; *P* = .98) (eFigure 3 in Supplement 1).

We also examined pooled rates according to the specific change outcome reported. Twenty-one studies^3,6,7,8,10,13,14,15,16,17,18,29,32,34,35,40,41,43,44^ reported RT plan changes (eFigure 4 in Supplement 1), and 14 studies^6,9,11,17,27,28,30,31,33,36,37,39,42,44^ reported recommendations for plan changes (eFigure 5 in Supplement 1); the pooled rates of each were 32.3% (95% CI, 20.7%-43.8%) and 22.4% (95% CI, 16.3%-28.5%), respectively. Among all 31 studies,^3,6,7,8,9,10,11,13,14,15,16,17,18,27,28,29,30,31,32,33,34,35,36,37,38,39,40,41,42,43,44^ the pooled rate of RT plan recommendations and changes was 29.0% (95% CI, 20.7%-37.2%) (eFigure 6 in Supplement 1).

### Subgroup Analyses of Plan Changes Based on PR and Study Factors

To better understand the association of the nature, frequency, and timing of PR with RT plan changes, subgroup analyses on the following factors were performed on all included studies: disease site and technique of RT plans being reviewed (all, head and neck, lung, SBRT of multiple sites, and other individual sites), frequency of PR (daily, multiple times per week, weekly, or as needed), and timing of PR relative to the RT planning process (before RT plan initiation or completion vs after or both). There were significant differences in RT plan changes between reports based on RT plan site and technique (eFigure 7 in Supplement 1), with studies reporting head and neck PR observing the highest rates of changes (44.9%; 95% CI, 31.3%-58.4%), followed by other sites (38.5%; 95% CI, −16.9% to 93.9%), lung (24.5%; 95% CI, 10.3%-37.8%), SBRT (17.4%; 95% CI, 2.7%-32.0%), and all or multiple sites (15.9%; 95% CI, 11.4%-20.4%) (*P* = .01). There were also significant differences in plan change rates based on the timing of PR (eFigure 8 in Supplement 1): studies reporting PR that was performed before RT planning observed a significantly higher rate (43.5%; 95% CI, 30.1%-56.8%) than those reporting PR after planning initiation or completion (11.4%; 95% CI, 6.9%-15.9%) or both before and after planning (21.0%; 95% CI, 15.8%-26.1%) (*P* < .001). There was no difference in change rates based on the frequency of PR (eFigure 9 in Supplement 1). When comparing studies reporting prospective vs retrospective analyses of PR outcomes, a significantly higher rate of changes was observed in prospective studies (36.7%; 95% CI, 25.4%-48.1%) than in retrospective studies (16.6%; 95% CI, 9.1%-24.2%) (*P* = .01) (eFigure 10 in Supplement 1). An association between radiologist involvement and plan changes was only observed in prospective studies (eFigures 11 and 12 in Supplement 1).

## Discussion

Curative RT is predicated on accurate, millimeter-precise treatment planning and delivery. Interdisciplinary collaboration between radiation oncologists and diagnostic radiologists may further refine this highly technical process, optimize RT targeting, and improve cancer treatment outcomes. In this systematic review and meta-analysis, we found a 36.8% higher absolute rate of clinically significant changes to the RT plan associated with radiologist inclusion in radiation oncology peer review of contoured volumes. Importantly, we did not observe a significant difference in minor changes, seemingly indicating that radiologist collaboration in PR meaningfully impacts RT plans without added change burden for minor issues. These findings are additionally important because PR is required for radiation oncology practice accreditation, and prior studies have shown RT plan quality directly affects cancer recurrence, progression, and survival.^45,46^

While multiple studies have been published on PR with or without radiology involvement, few have compared PR outcomes between the 2 approaches. Only one of these experiences involved a prospective clinical trial that specifically investigated radiation oncology–radiology collaboration as a treatment planning intervention for patients with locally advanced lung cancer: addition of a radiologist to the PR process resulted in changes in the tumor volume in over one-third of patients.^17^ Among patients with head and neck cancer, Gatfield et al^18^ prospectively compared PR outcomes with vs without radiology involvement and found that the presence of a neuroradiologist increased the rate of changes to delineated structures by two-thirds (from 33% to 55%). While changes to RT targets or OARs may increase with additional layers of PR, it is yet to be determined whether these changes result in different clinical outcomes. One study reporting head and neck RT PR identified a mean volumetric change in target volumes of 20% to 22%, with the upper range being greater than 100% to greater than 275%.^14^ These quantities represent substantial revisions to the RT targets and, in the era of highly conformal RT techniques, could be expected to impact the risk of locoregional failure.

The benefits of radiology involvement in PR of radiation oncologist–delineated contours must outweigh the potential barriers to routine implementation of this practice. The main potential benefit apparent from our meta-analysis is clinically meaningful improvements to the RT plan. However, barriers include time limitations, technological considerations, and lack of a current reimbursement model for this additional effort. Radiation oncologists may worry that radiologist participation in PR could elicit additional time-consuming but less clinically meaningful minor changes, though the findings from our meta-analysis do not support an increase in minor change rates. Time constraints on both sides of the interdisciplinary spectrum must be considered: attending PR conferences or soliciting on-demand PR during busy clinical services is a major barrier. The most efficient PR method may vary by institution; the logistics of radiology–radiation oncology PR may include in-person or virtual conferences, telephone conversations, asynchronous review of images via the electronic health record, or other methods of communication. Novel methods to facilitate interdisciplinary review of RT targets that improve the ease of use for the reviewing radiologist are needed. Farris et al^17^ developed a novel script-based framework for transferring the contoured images from the RT planning computed tomographic image set into an anonymized portable document format (PDF) that can then be shared with the reviewing radiologist. Development of automated systems integrated within the institutional picture archiving and communication systems may increase efficiency, improve implementation, and inform follow-up imaging interpretation.^47,48^ However, without a reimbursement mechanism, radiologists’ time spent reviewing radiation oncologists’ targets ultimately detracts from their diagnostic productivity, which may require up to 30 to 60 minutes per case review, depending on the complexity.^17^ Considering the high cost of salvage or palliative therapies (such as immunotherapy) for patients with recurrence after RT and the high rates of clinically significant changes to the RT plan identified in our study, preventing failure through radiologist PR could represent a cost-effective intervention. Prospective evidence of improved oncologic outcomes would be necessary to support the development of professional reimbursement mechanisms or procedure codes like those used in other RT planning procedures, such as radiosurgery.

Our findings also provide practical insight into the efficient and cost-effective use of multidisciplinary resources for collaborative PR of RT targets. Subgroup analyses indicate that radiology incorporation into PR of head and neck and lung cancer treatment plans yields consistently high rates of change. We also observed significantly higher change rates to the RT plan when PR is performed before compared with after the RT plan has already been finalized. Since there was no difference in the rate of RT plan changes based on frequency of PR, the most flexible and feasible schedule that maximizes interdisciplinary participation can be used without the risk of affecting PR outcomes. A streamlined process for radiation oncology–radiology collaboration may also increase the efficiency and quality of image interpretation after RT, as the details of RT location, distribution, and timing are often not readily available in the medical record.^49^

### Limitations

This study is limited by the study-level nature of the meta-analysis, the lack of studies reporting PR outcomes with and without radiologist involvement, the imbalance in total number of cases between groups, and the high heterogeneity of the included studies. Due to the small number of cases with radiology-based PR, these findings should be interpreted with caution. The fact that 0.6% of all identified cases (1.2% when excluding the 20 069 cases reported in the largest nonradiology study by Tchelebi et al^10^) reported radiology-based PR highlights the critical need for additional data in this space. Despite the ubiquity of PR in radiation oncology and routine collaboration between radiation oncology and radiology in the course of clinical care, limited data exist to measure the clinical impact of radiologist–radiation oncologist collaboration in PR of contoured targets. Additional prospective investigations including multiple disease sites are necessary to better understand the clinical impact of incorporating radiologists in radiation oncology PR in an unbiased, unselected sample. While there are differences in sample size between groups, meta-analytic techniques, such as the random-effects models reported herein, are expected to minimize confounding. Regarding heterogeneity, while removal of outlier studies in the primary analyses of all changes, major changes, and minor changes reduced heterogeneity to a reasonable level (*I*^2^ < 50%) in the radiologist group, heterogeneity remained high in the group without a radiologist. Since PR in radiation oncology is highly variable by nature, this heterogeneity is expected and suggests generalizability across multiple PR methods, timing, and formats. Additionally, removal of outliers did not substantially change the findings of these analyses. Because only 1 study reported outcomes of PR with and without radiologist involvement, comparisons within studies were not possible, so the findings of these comparisons between studies are subject to confounding. The inclusion of retrospective and prospective studies may also introduce bias, as a higher rate of changes was observed in prospective studies, as would be expected in quality improvement studies aimed at improving RT plans through prospective assessment of PR. Additional prospective study of this interdisciplinary collaboration can investigate the impact of radiology involvement directly on clinical outcomes, such as local disease control and survival.

## Conclusions

In this systematic review and meta-analysis of radiation oncology PR of contoured targets, radiologist involvement was associated with higher rates of clinically relevant changes to the RT target and total changes to the RT plan. Radiologist involvement was not associated with rates of minor RT plan changes. These results support the value of interdisciplinary collaboration with radiology during RT planning. Further efforts to maximize the efficiency and cost-effectiveness of closer interdisciplinary collaboration between these 2 specialties would be expected to increase the quality of RT and improve cancer outcomes, which warrants evaluation in prospective clinical trials.

## Notes

Supplement 1. **eMethods.** Search Strategy **eFigure 1.** PRISMA Flow Diagram **eFigure 2.** Funnel Plot for Publication Bias of RT Plan Changes Based on Sample Size in All Included Studies **eFigure 3.** Forest Plot and Pooled Rates of Changes to Organs at Risk by Radiology Involvement **eFigure 4.** Forest Plot and Pooled Estimates of Changes to the Radiotherapy Plan as a Result of Peer Review **eFigure 5.** Forest Plot and Pooled Estimates of Recommendations for Changes to the Radiotherapy Plan as a Result of Peer Review **eFigure 6.** Forest Plot and Pooled Estimates of Plan Changes and Recommendations for Change **eFigure 7.** Forest Plot and Pooled Estimates of Plan Changes Based on Cancer Site **eFigure 8.** Forest Plot and Pooled Estimates of Plan Changes Based on Timing of Peer Review Related to the RT Planning Process **eFigure 9.** Forest Plot and Pooled Estimates of Plan Changes Based on Frequency of Peer Review Meetings **eFigure 10.** Forest Plot and Pooled Estimates of Plan Changes in Prospective vs Retrospective Studies **eFigure 11.** Forest Plot and Pooled Estimates of Plan Changes by Radiology Involvement in a Subgroup Analysis of Only Prospective Studies **eFigure 12.** Forest Plot and Pooled Estimates of Plan Changes Based on Radiology Involvement in a Subgroup Analysis of Only Retrospective Studies **eTable 1.** Characteristics of Included Studies **eTable 2.** Intent of Treatment **eTable 3.** Quality of Included Studies Assessed by the MINORS Criteria

Supplement 2.
Data Sharing Statement
