# Supplementary material for: Radiologist Involvement in Radiation Oncology Peer Review: A Systematic Review and Meta-Analysis
Source: JAMA Netw Open. 2024 Dec 27;7(12):e2452667. doi: 10.1001/jamanetworkopen.2024.52667 (PMC11681381; doi:10.1001/jamanetworkopen.2024.52667)
Supplement: Supplement 2. — Data Sharing Statement [file jamanetwopen-e2452667-s002.pdf]

Data Sharing Statement

Hughes. Radiologist Involvement in Radiation Oncology Peer Review. *JAMA Netw Open*. Published December 27, 2024. doi:10.1001/jamanetworkopen.2024.52667

Data
Data available: Yes
Data types: Data (not involving human participants)
How to access data: ryhughes@wakehealth.edu
When available: With publication

Supporting Documents
Document types: None

Additional Information
Who can access the data: anyone requesting the data
Types of analyses: for research purposes
Mechanisms of data availability: upon formal request to the corresponding author
