# Supplementary material for: Radiologist Involvement in Radiation Oncology Peer Review: A Systematic Review and Meta-Analysis
Source: JAMA Netw Open. 2024 Dec 27;7(12):e2452667. doi: 10.1001/jamanetworkopen.2024.52667 (PMC11681381; doi:10.1001/jamanetworkopen.2024.52667)
Supplement: Supplement 1. — eMethods. Search Strategy eFigure 1. PRISMA Flow Diagram eFigure 2. Funnel Plot for Publication Bias of RT Plan Changes Based on Sample Size in All Included Studies eFigure 3. Forest Plot and Pooled Rates of Changes to Organs at Risk by Radiology Involvement eFigure 4. Forest Plot and Pooled Estimates of Changes to the Radiotherapy Plan as a Result of Peer Review eFigure 5. Forest Plot and Pooled Estimates of Recommendations for Changes to the Radiotherapy Plan as a Result of Peer Review eFigure 6. Forest Plot and Pooled Estimates of Plan Changes and Recommendations for Change eFigure 7. Forest Plot and Pooled Estimates of Plan Changes Based on Cancer Site eFigure 8. Forest Plot and Pooled Estimates of Plan Changes Based on Timing of Peer Review Related to the RT Planning Process eFigure 9. Forest Plot and Pooled Estimates of Plan Changes Based on Frequency of Peer Review Meetings eFigure 10. Forest Plot and Pooled Estimates of Plan Changes in Prospective vs Retrospective Studies eFigure 11. Forest Plot and Pooled Estimates of Plan Changes by Radiology Involvement in a Subgroup Analysis of Only Prospective Studies eFigure 12. Forest Plot and Pooled Estimates of Plan Changes Based on Radiology Involvement in a Subgroup Analysis of Only Retrospective Studies eTable 1. Characteristics of Included Studies eTable 2. Intent of Treatment eTable 3. Quality of Included Studies Assessed by the MINORS Criteria [file jamanetwopen-e2452667-s001.pdf]

Supplementary Online Content

Hughes RT, Razavian NB, Smith S, et al. Radiologist involvement in radiation oncology peer review: a systematic review and meta-analysis. *JAMA Netw Open*. 2024;7(12):e2452667. doi:10.1001/jamanetworkopen.2024.52667

eMethods. Search Strategy

eFigure 1. PRISMA Flow Diagram

eFigure 2. Funnel Plot for Publication Bias of RT Plan Changes Based on Sample Size in All Included Studies

eFigure 3. Forest Plot and Pooled Rates of Changes to Organs at Risk by Radiology Involvement

eFigure 4. Forest Plot and Pooled Estimates of Changes to the Radiotherapy Plan as a Result of Peer Review

eFigure 5. Forest Plot and Pooled Estimates of Recommendations for Changes to the Radiotherapy Plan as a Result of Peer Review

eFigure 6. Forest Plot and Pooled Estimates of Plan Changes and Recommendations for Change

eFigure 7. Forest Plot and Pooled Estimates of Plan Changes Based on Cancer Site

eFigure 8. Forest Plot and Pooled Estimates of Plan Changes Based on Timing of Peer Review Related to the RT Planning Process

eFigure 9. Forest Plot and Pooled Estimates of Plan Changes Based on Frequency of Peer Review Meetings

eFigure 10. Forest Plot and Pooled Estimates of Plan Changes in Prospective vs Retrospective Studies

eFigure 11. Forest Plot and Pooled Estimates of Plan Changes by Radiology Involvement in a Subgroup Analysis of Only Prospective Studies

eFigure 12. Forest Plot and Pooled Estimates of Plan Changes Based on Radiology Involvement in a Subgroup Analysis of Only Retrospective Studies

eTable 1. Characteristics of Included Studies

eTable 2. Intent of Treatment

eTable 3. Quality of Included Studies Assessed by the MINORS Criteria

This supplementary material has been provided by the authors to give readers additional information about their work.

© 2024 Hughes RT et al. *JAMA Network Open*.

eMethods. Search Strategy

Three electronic databases (PubMed, Embase, and Web of Science) were queried for published articles up to March 6, 2024. The following search terms were used:

("quality assurance" OR "peer review" OR “chart rounds”) AND ("contour" OR "contours" OR “contouring” OR "segmented" OR "delineated" OR "delineation" OR "target" OR "targets" OR “volume” OR “volume delineation”) AND ("radiotherapy" OR "radiation" OR "radiation therapy")

Titles and abstracts were screened by two reviewers (R.T.H. and M.K.F.). In the case of discordance, discussion was used to reach consensus regarding inclusion for full-text review. To reduce the risk of bias, case reports and studies reporting the PR of less than 10 patients were excluded. When studies included overlapping patient populations from the same institution, the most recent study was included.

Articles were included if they reported peer review (either chart rounds or prospective peer review) of radiotherapy contours with or without radiology involvement. Articles were reviewed by 2 or more authors and discrepancies were resolved by consensus. Studies excluded from final analysis were: available in abstract only, foreign language, studies reporting artificial intelligence- or machine learning-based peer review, case reports or case series with <10 cases, focused on clinical trial RT plan QA or benchmarking, database studies (SEER/NCDB), dosimetric planning studies, quality improvement studies, peer review implementation studies, studies focused on non-clinical physics or patient specific QA topics, studies without patients treated with external beam radiotherapy (e.g., brachytherapy, stereotactic radiosurgery only), guideline/white paper articles, those outside of the scope of peer review of contoured radiotherapy targets, studies ongoing, review articles, those with overlapping patient populations, and systematic reviews or meta-analyses.

© 2024 Hughes RT et al. *JAMA Network Open*.

eFigure 1. PRISMA Flow Diagram

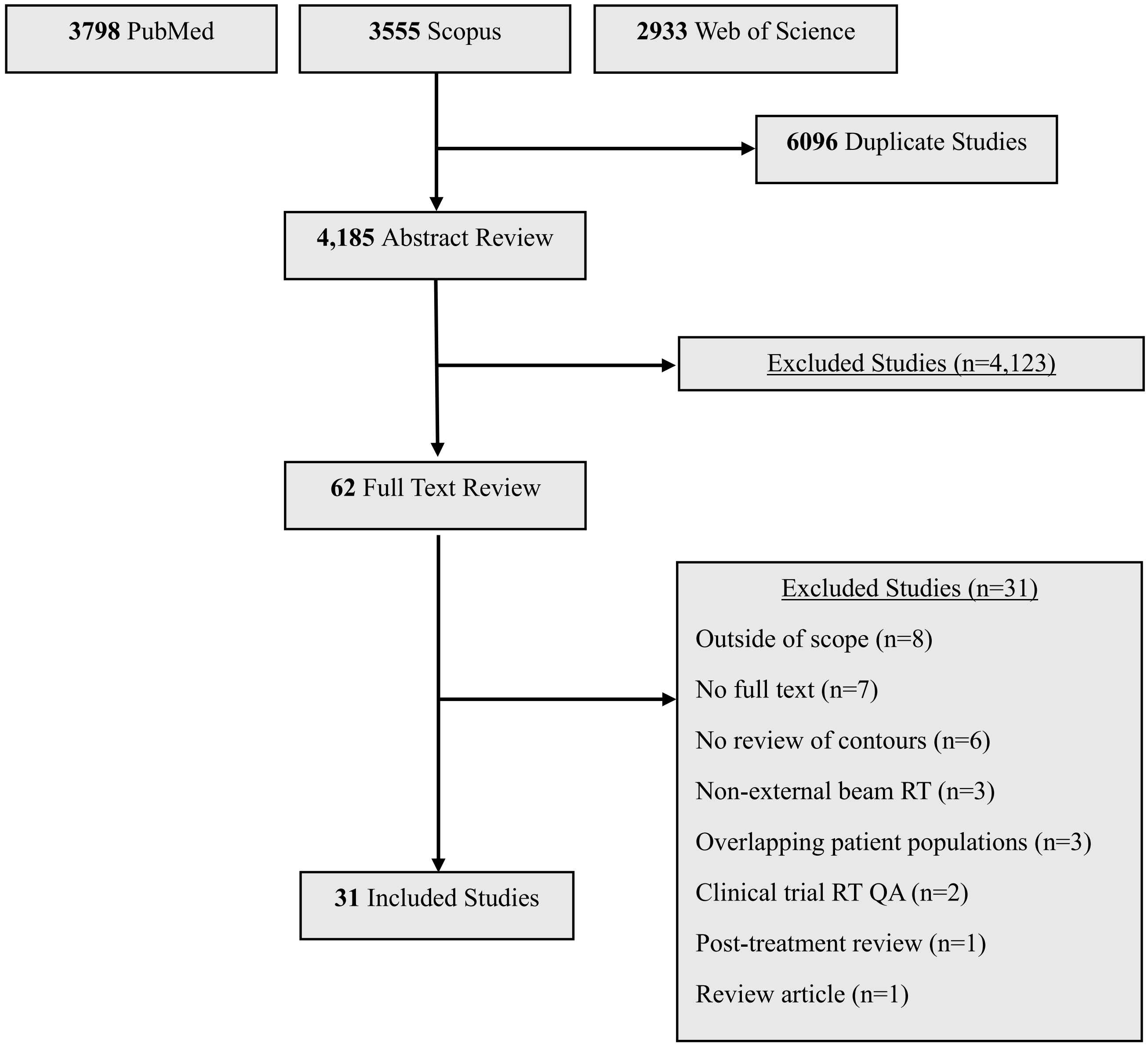

© 2024 Hughes RT et al. *JAMA Network Open*.

eFigure 2. Funnel Plot for Publication Bias of RT Plan Changes Based on Sample Size in All Included Studies

The Egger's regression test P = 0.56. A P value greater than 0.05 indicates no statistically significant publication bias identified.

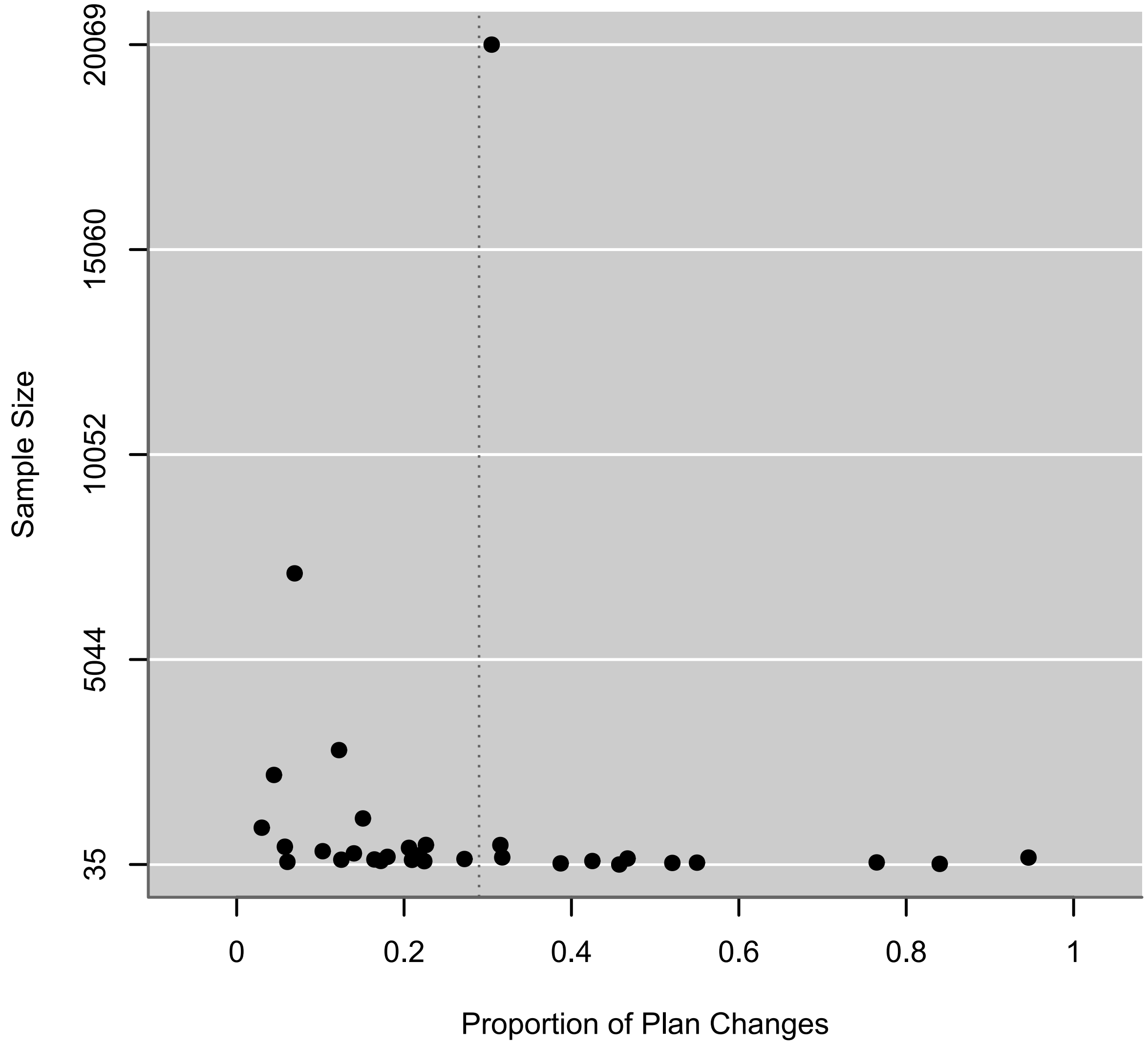

© 2024 Hughes RT et al. *JAMA Network Open*.

eFigure 3. Forest Plot and Pooled Rates of Changes to Organs at Risk by Radiology Involvement

For the one study that included two cohorts undergoing PR with and without radiology, Gatfield et al., 2023 is reproduced twice. Gatfield, 2023.2 indicates the group reviewed without a radiologist, Gatfield, 2023.1 indicates the group reviewed with a radiologist.

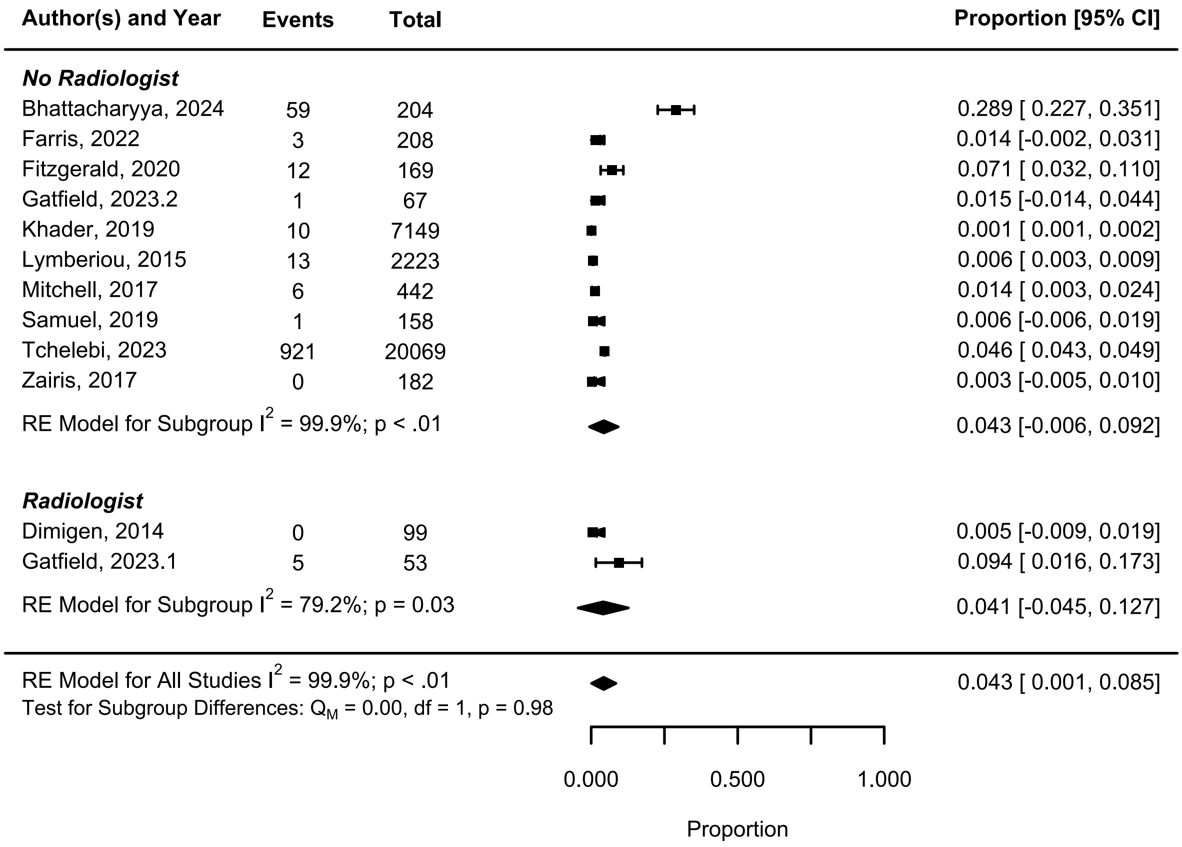

© 2024 Hughes RT et al. *JAMA Network Open*.

eFigure 4. Forest Plot and Pooled Estimates of Changes to the Radiotherapy Plan as a Result of Peer Review

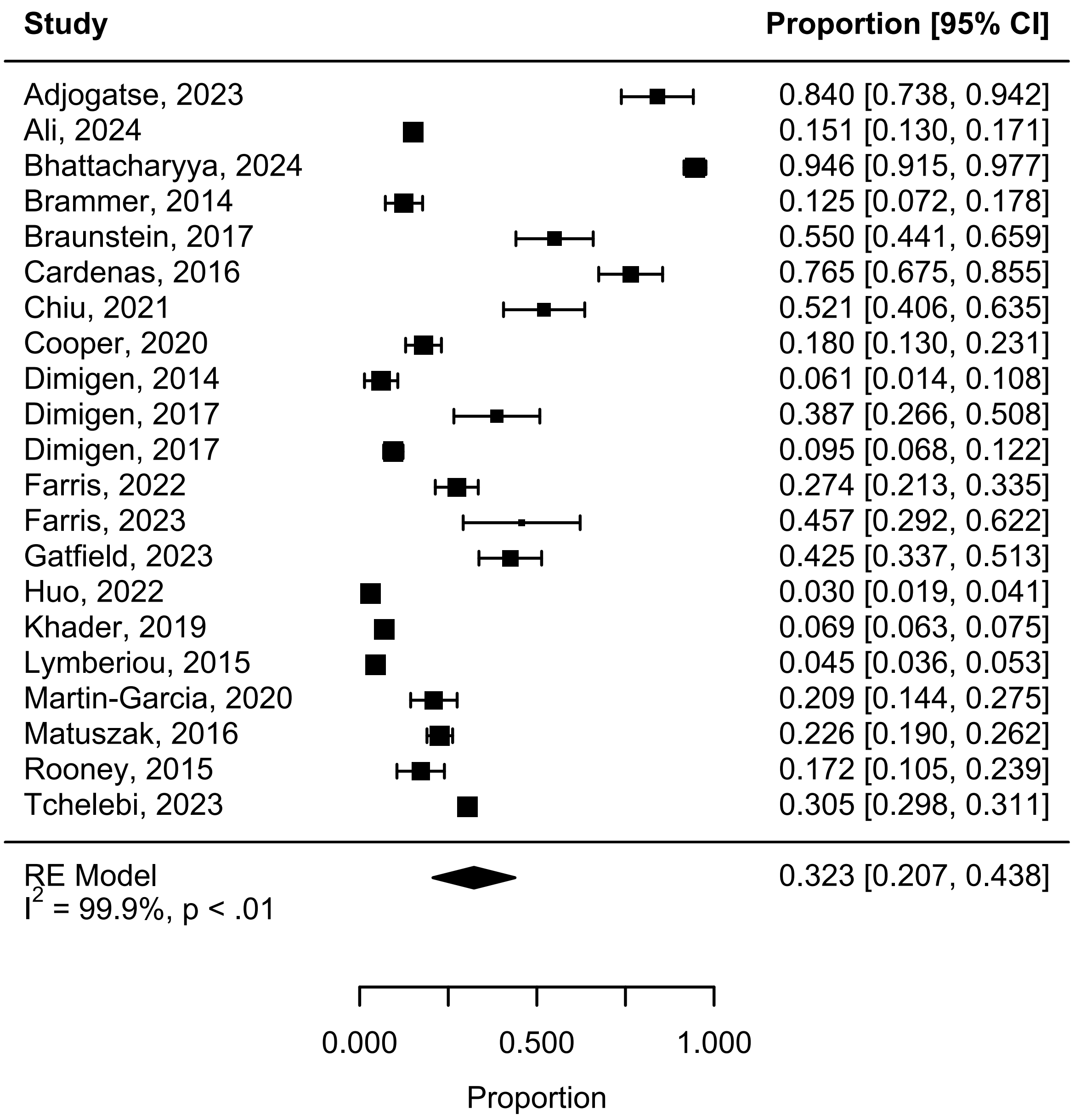

© 2024 Hughes RT et al. *JAMA Network Open*.

eFigure 5. Forest Plot and Pooled Estimates of Recommendations for Changes to the Radiotherapy Plan as a Result of Peer Review

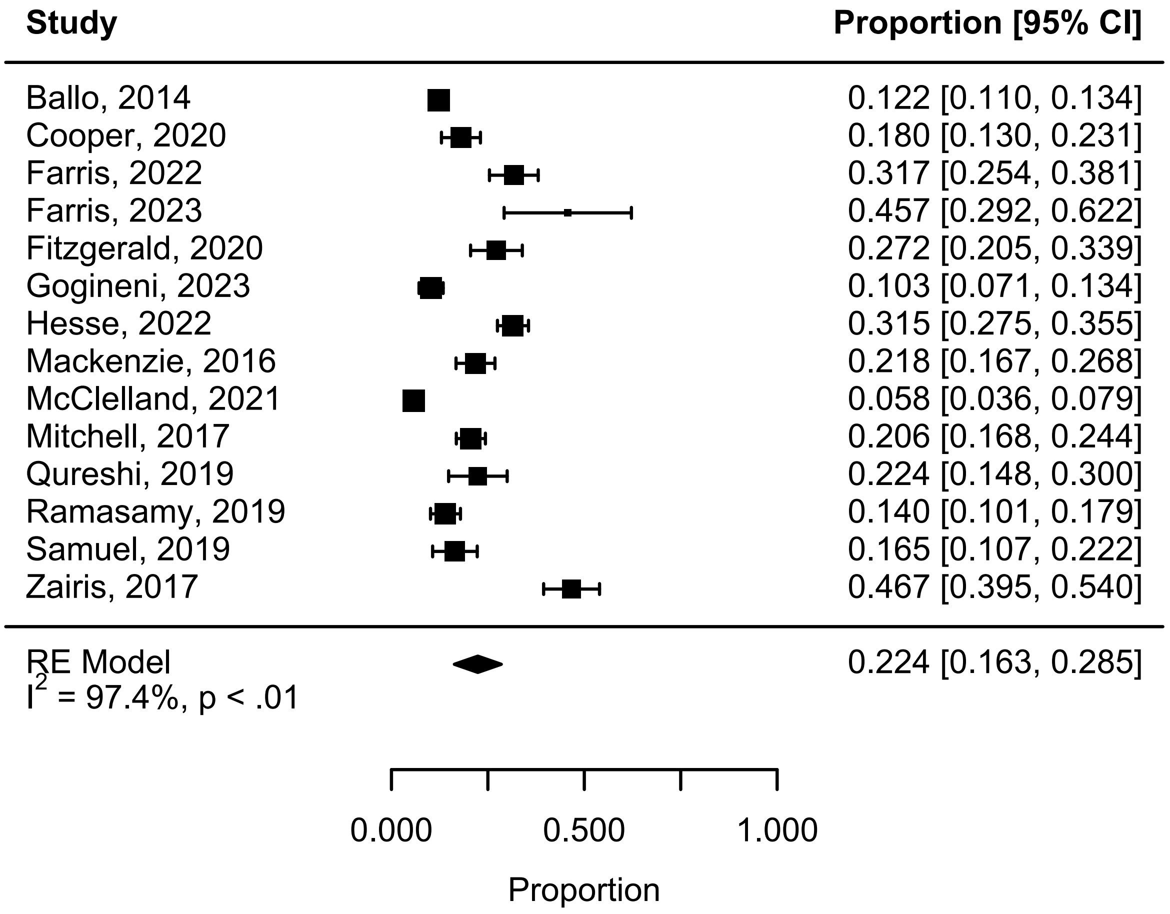

© 2024 Hughes RT et al. *JAMA Network Open*.

eFigure 6. Forest Plot and Pooled Estimates of Plan Changes and Recommendations for Change

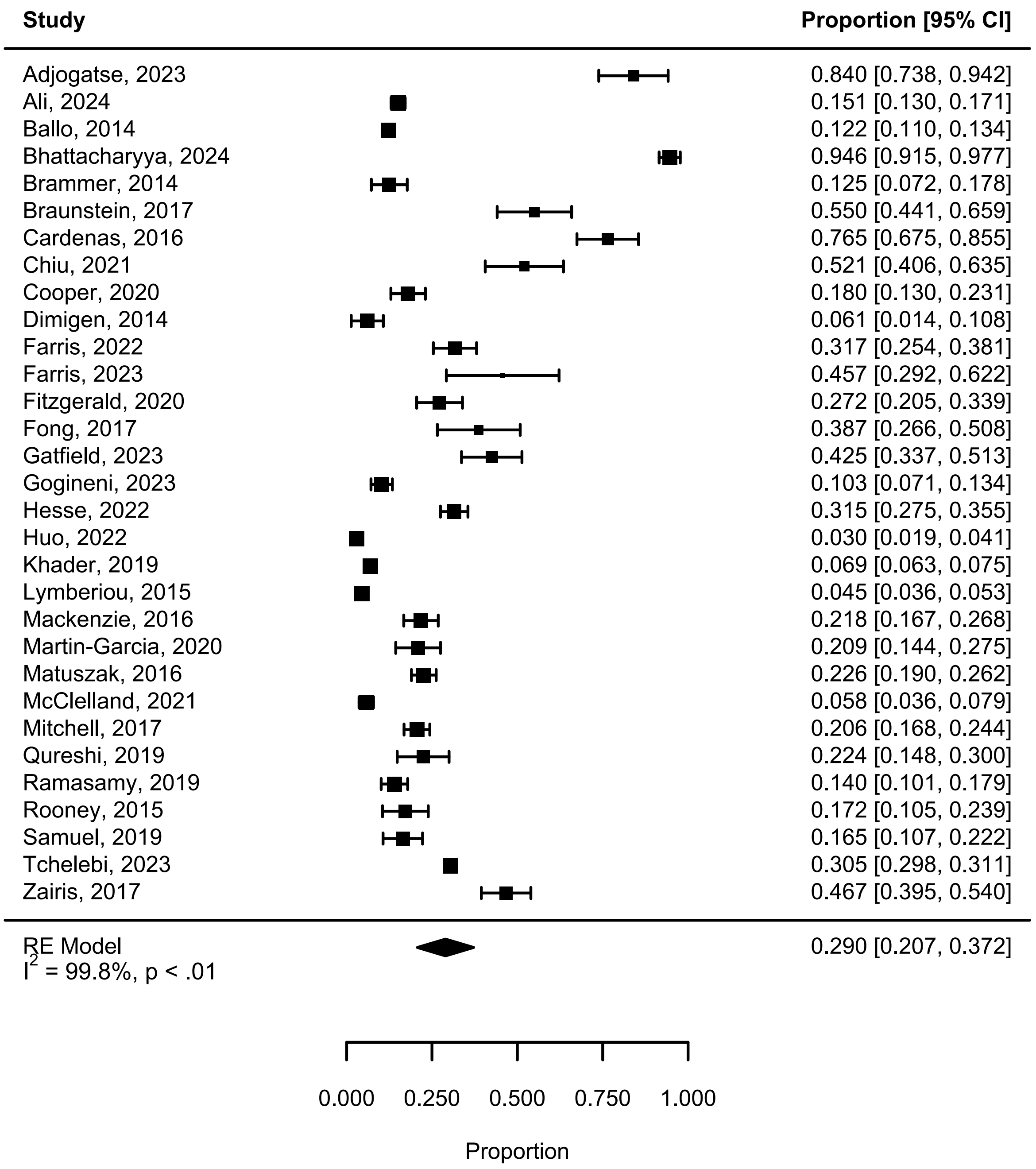

© 2024 Hughes RT et al. *JAMA Network Open*.

eFigure 7. Forest Plot and Pooled Estimates of Plan Changes Based on Cancer Site

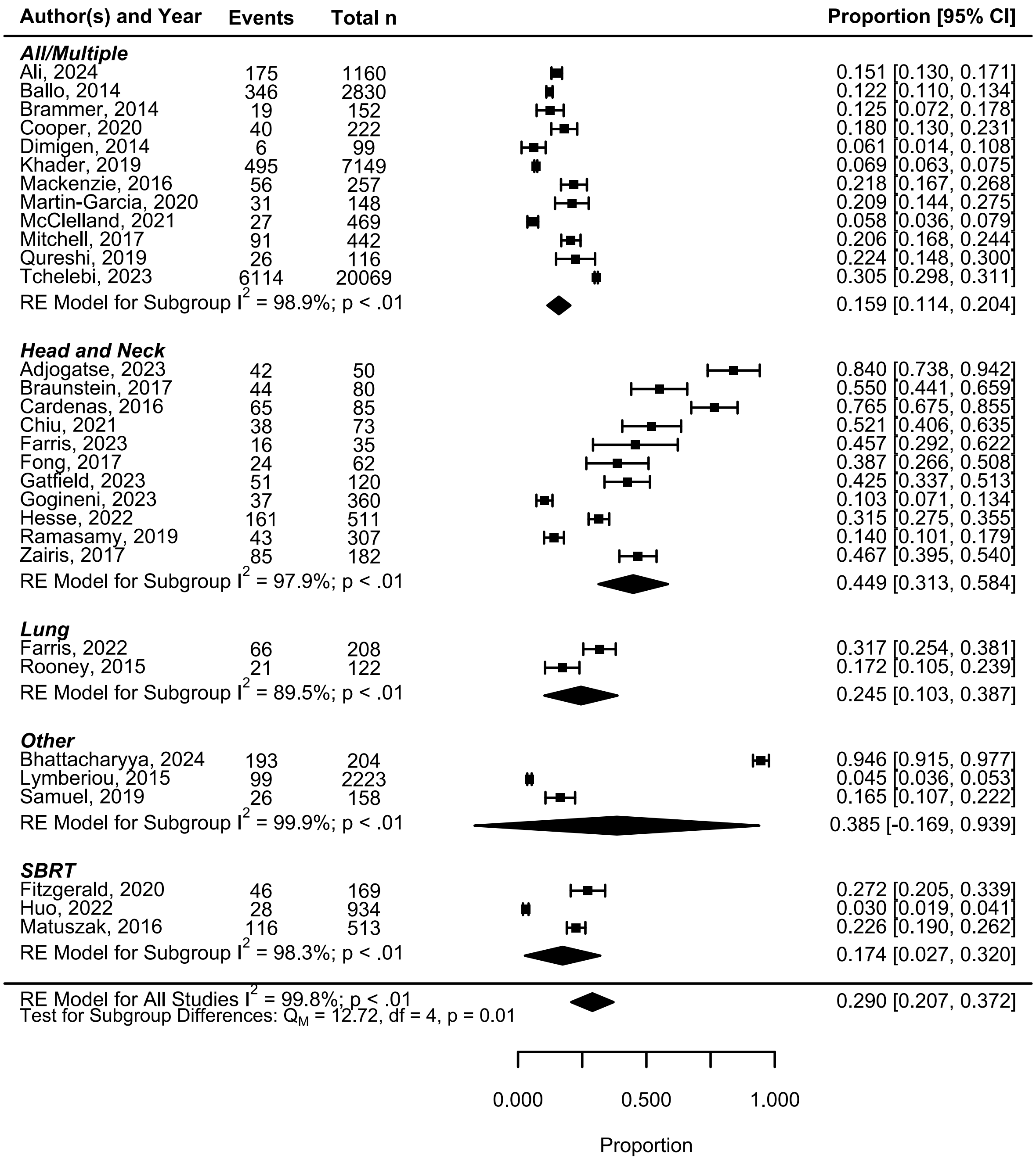

© 2024 Hughes RT et al. *JAMA Network Open*.

eFigure 8. Forest Plot and Pooled Estimates of Plan Changes Based on Timing of Peer Review Related to the RT Planning Process

Author(s) and Year	Events	Total n	Proportion [95% CI]
Pre-planning			
Adjogatse, 2023	42	50	0.840 [0.738, 0.942]
Bhattacharyya, 2024	193	204	0.946 [0.915, 0.977]
Braunstein, 2017	44	80	0.550 [0.441, 0.659]
Cardenas, 2016	65	85	0.765 [0.675, 0.855]
Chiu, 2021	38	73	0.521 [0.406, 0.635]
Dimigen, 2014	6	99	0.061 [0.014, 0.108]
Farris, 2022	66	208	0.317 [0.254, 0.381]
Farris, 2023	16	35	0.457 [0.292, 0.622]
Fong, 2017	24	62	0.387 [0.266, 0.508]
Gatfield, 2023	51	120	0.425 [0.337, 0.513]
Gogineni, 2023	37	360	0.103 [0.071, 0.134]
Hesse, 2022	161	511	0.315 [0.275, 0.355]
Matuszak, 2016	116	513	0.226 [0.190, 0.262]
Samuel, 2019	26	158	0.165 [0.107, 0.222]
Zairis, 2017	85	182	0.467 [0.395, 0.540]
RE Model for Subgroup I^2 = 98.8%; p < .01			0.435 [0.301, 0.568]
Post-planning			
Ali, 2024	175	1160	0.151 [0.130, 0.171]
Ballo, 2014	346	2830	0.122 [0.110, 0.134]
Huo, 2022	28	934	0.030 [0.019, 0.041]
Khader, 2019	495	7149	0.069 [0.063, 0.075]
Lymberiou, 2015	99	2223	0.045 [0.036, 0.053]
Martin-Garcia, 2020	31	148	0.209 [0.144, 0.275]
McClelland, 2021	27	469	0.058 [0.036, 0.079]
Mitchell, 2017	91	442	0.206 [0.168, 0.244]
Rooney, 2015	21	122	0.172 [0.105, 0.239]
RE Model for Subgroup I^2 = 99.0%; p < .01			0.114 [0.069, 0.159]
Both Pre- and Post-planning			
Brammer, 2014	19	152	0.125 [0.072, 0.178]
Cooper, 2020	40	222	0.180 [0.130, 0.231]
Fitzgerald, 2020	46	169	0.272 [0.205, 0.339]
Mackenzie, 2016	56	257	0.218 [0.167, 0.268]
Qureshi, 2019	26	116	0.224 [0.148, 0.300]
Ramasamy, 2019	43	307	0.140 [0.101, 0.179]
Tchelebi, 2023	6114	20069	0.305 [0.298, 0.311]
RE Model for Subgroup I^2 = 91.8%; p < .01			0.210 [0.158, 0.261]
RE Model for All Studies I^2 = 99.8%; p < .01			0.290 [0.207, 0.372]

Test for Subgroup Differences: Q_M = 16.93, df = 2, p < .01

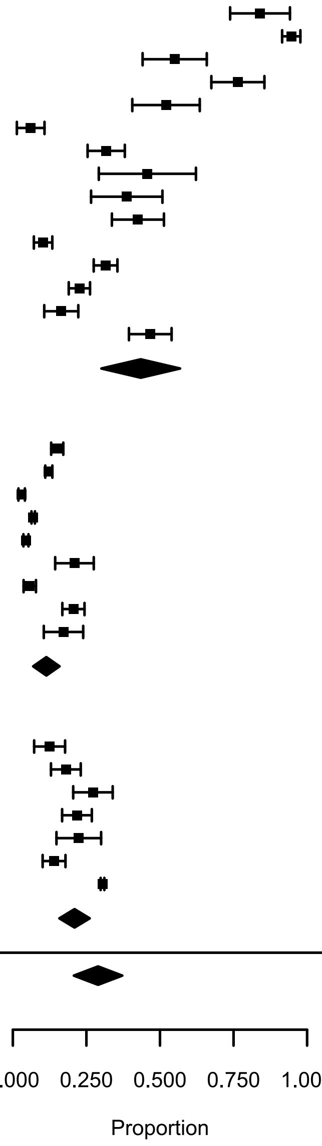

© 2024 Hughes RT et al. *JAMA Network Open*.

eFigure 9. Forest Plot and Pooled Estimates of Plan Changes Based on Frequency of Peer Review Meetings

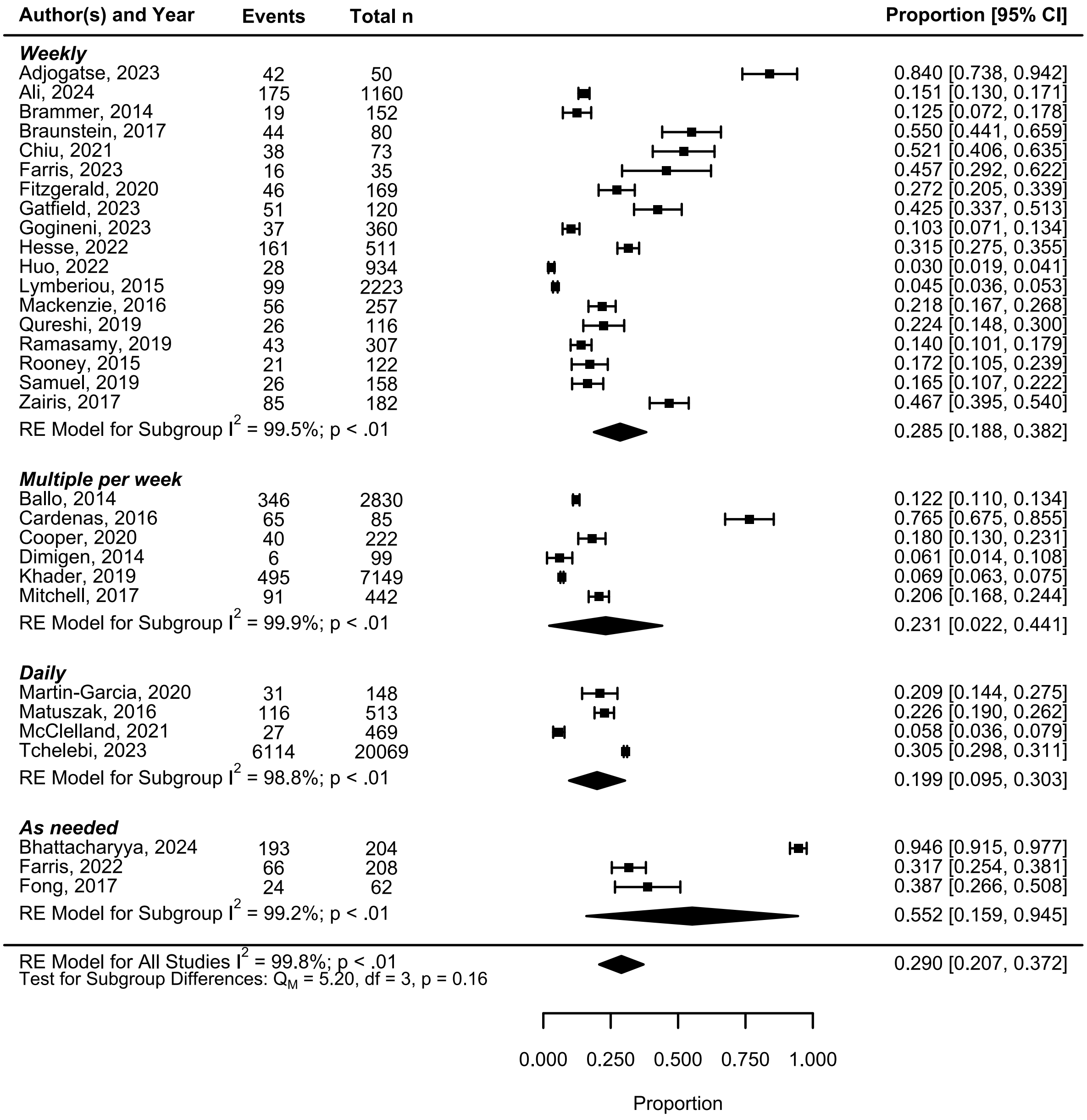

© 2024 Hughes RT et al. *JAMA Network Open*.

eFigure 10. Forest Plot and Pooled Estimates of Plan Changes in Prospective vs Retrospective Studies

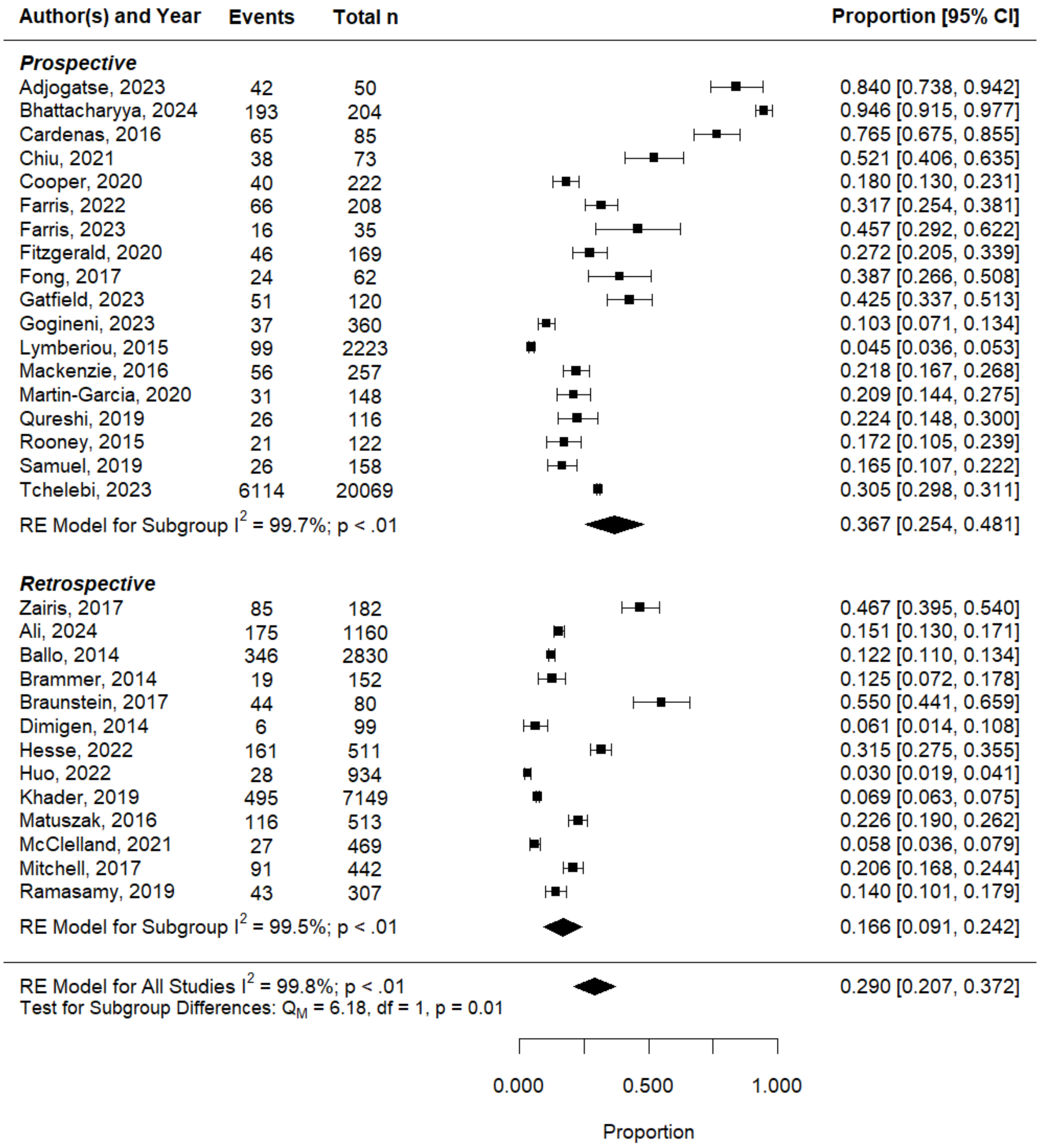

© 2024 Hughes RT et al. *JAMA Network Open*.

eFigure 11. Forest Plot and Pooled Estimates of Plan Changes by Radiology Involvement in a Subgroup Analysis of Only Prospective Studies

For the one study that included two cohorts undergoing PR with and without radiology, Gatfield et al., 2023 is reproduced twice. Gatfield, 2023.2 indicates the group reviewed without a radiologist, Gatfield, 2023.1 indicates the group reviewed with a radiologist.

Author(s) and Year	Events	Total n	Proportion [95% CI]
No Radiologist			
Bhattacharyya, 2024	193	204	0.946 [0.915, 0.977]
Cardenas, 2016	65	85	0.765 [0.675, 0.855]
Cooper, 2020	40	222	0.180 [0.130, 0.231]
Farris, 2022	66	208	0.317 [0.254, 0.381]
Fitzgerald, 2020	46	169	0.272 [0.205, 0.339]
Fong, 2017	24	62	0.387 [0.266, 0.508]
Gatfield, 2023.2	22	67	0.328 [0.216, 0.441]
Gogineni, 2023	37	360	0.103 [0.071, 0.134]
Lymberiou, 2015	99	2223	0.045 [0.036, 0.053]
Mackenzie, 2016	56	257	0.218 [0.167, 0.268]
Martin-Garcia, 2020	31	148	0.209 [0.144, 0.275]
Qureshi, 2019	26	116	0.224 [0.148, 0.300]
Rooney, 2015	21	122	0.172 [0.105, 0.239]
Samuel, 2019	26	158	0.165 [0.107, 0.222]
Tchelebi, 2023	6114	20069	0.305 [0.298, 0.311]
Zairis, 2017	85	182	0.467 [0.395, 0.540]
RE Model for Subgroup I^2 = 99.7%; p < .01			0.318 [0.201, 0.435]
Radiologist			
Adjogatse, 2023	42	50	0.840 [0.738, 0.942]
Chiu, 2021	38	73	0.521 [0.406, 0.635]
Farris, 2023	16	35	0.457 [0.292, 0.622]
Gatfield, 2023.1	29	53	0.547 [0.413, 0.681]
RE Model for Subgroup I^2 = 86.5%; p < .01			0.597 [0.426, 0.768]
RE Model for All Studies I^2 = 99.7%; p < .01			0.371 [0.262, 0.481]

Test for Subgroup Differences: Q_M = 4.46, df = 1, p = 0.03

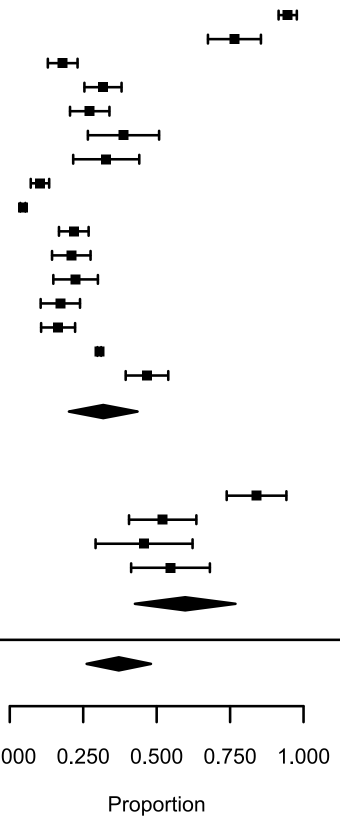

© 2024 Hughes RT et al. *JAMA Network Open*.

eFigure 12. Forest Plot and Pooled Estimates of Plan Changes Based on Radiology Involvement in a Subgroup Analysis of Only Retrospective Studies

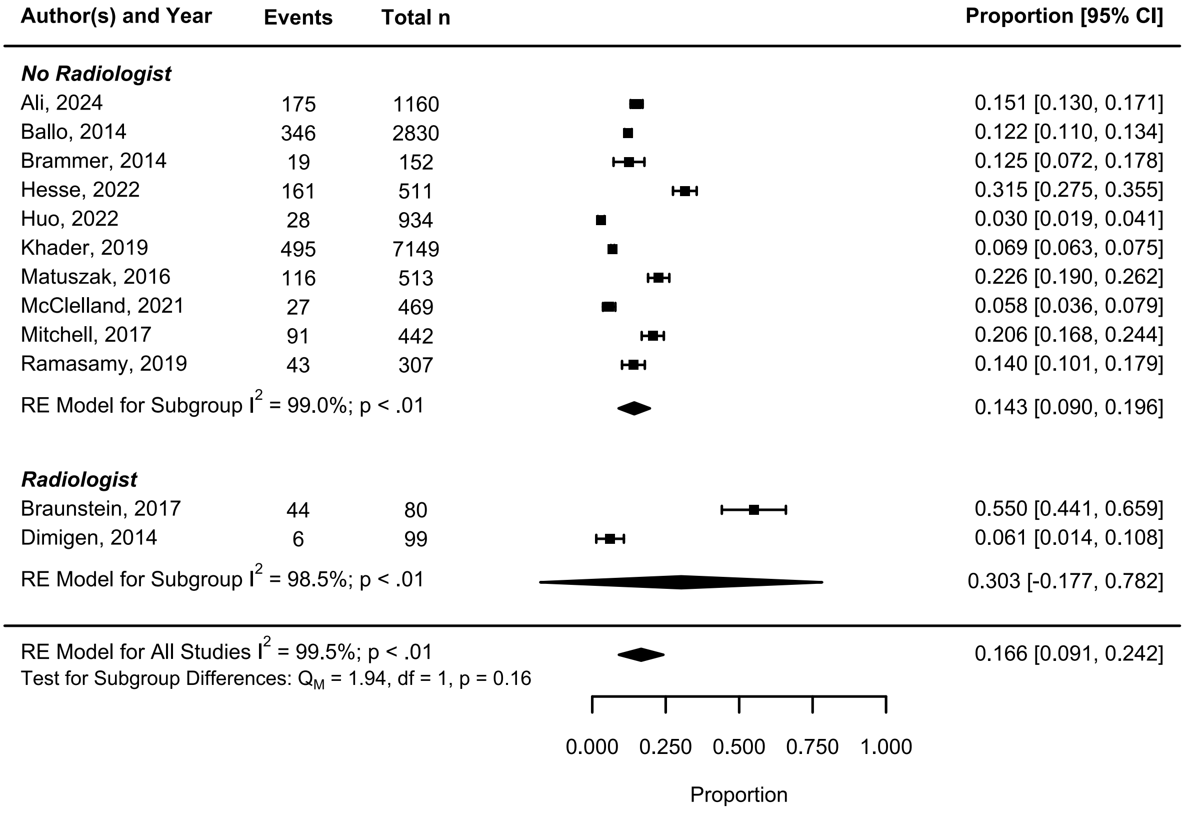

© 2024 Hughes RT et al. *JAMA Network Open*.

eTable 1. Characteristics of Included Studies

Study	Year	Design	Country	Disease Site(s)	Frequency	Radiology	Structures Reviewed	Change Severity Assessment	Definition of Major Change	Definition of Minor Change
Adjogatse	2023	P	UK	HN	Weekly	Y	GTV	Clinically significant; clinically nonsignificant	Having a clinically significant alteration per delineating radiation oncologist	Does not meet the criteria for significant
Ali	2024	R	US	All	Weekly	N	All	Major; minor	Treatment should be stopped until peer-review feedback is addressed	May be concerns with the plan, however the treatment does not need to be urgently stopped to address these
Ballo	2014	R	US	All	Twice weekly	N	All	Major; minor	Changed treatment modality, added modalities	Changes to radiation dose or target volume
Bhattacharyya	2024	P	India	GYN	As needed	N	Targets	Major; minor	Change is done to prevent geographical miss of the tumor, change in contour in any direction >1 cm, or that is likely to affect the likelihood of locoregional control	Change requiring editing of a contour of 1 cm in any direction or and that is unlikely to affect the likelihood of locoregional tumor control

© 2024 Hughes RT et al. *JAMA Network Open*.

Brammer	2014	R	UK	All	Weekly	N	CTV	Major; minor	Any alterations that required a change in the delivery of radiotherapy	Alterations that did not require a physical alteration of the treatment plan (i.e., documentation or labelling)
Braunstein	2017	R	US	HN	Weekly	Y	GTV, CTV	Clinically significant; clinically nonsignificant	Resulted in exclusion or inclusion of a distinct area or structure and would change the RT plan with potential impact on disease control or toxicity	Does not meet the criteria for significant
Cardenas	2016	P	US	HN	Biweekly	N	Targets, OARs	Major; minor	Clinically affect the likelihood of cure, adverse events, or locoregional control	Elective or stylistic
Chiu	2021	P	UK	HN	Weekly	Y	GTV, CTV	Major; minor	Required alteration of the plan to prevent a geographical miss, or a change in the treatment paradigm	Change without which the original volumes would still have been deemed clinically acceptable

© 2024 Hughes RT et al. *JAMA Network Open*.

Cooper	2020	P	US	All	Biweekly	N	GTV, CTV, OARs	None	NA	NA
Dimigen	2014	R	Australia	All	Biweekly	Y	GTV, CTV	None	NA	NA
Farris	2023	P	US	LU	On demand	Y	GTV	None	NA	NA
Farris	2022	P	US	HN	Weekly	N	GTV, CTV, PTV, OARs	Major; minor	Modification to any one of the following: GTV, HR-PTV, Rx/fractionation	Modification to any one of the following: IR-PTV, LR-PTV, any OAR
Fitzgerald	2020	P	Australia	SBRT	Weekly	N	Targets, OARs	None	NA	NA
Fong	2017	P	UK	HN	On demand	N	Targets	Significant; minor	High risk of potential geographical miss or extensive unnecessary impact on critical organs at risk or normal tissues	Change, but original would have been acceptable
Gatfield	2023	P	UK	HN	Weekly	Y (44%), N (56%)	All	Major; minor	Alteration to the GTV or high-dose CTV	Alteration to the elective CTV
Gogineni	2023	P	US	HN	Weekly	N	PTV, OARs	Red; yellow	Major change that was reserved for volumes that did not reflect the standard of care and were deemed to have significant potential to	Minor modifications, such as a small adjustment to the target volumes, alternative suggestion for margins, or

© 2024 Hughes RT et al. *JAMA Network Open*.

									impact likelihood of control, survival, or toxicity, such as under covered areas of gross or microscopic disease within the PTV	changing the dose fractionation schedule. These requested changes were considered elective or stylistic
Hesse	2022	R	US	HN	Weekly	N	Targets	Major; minor	significant changes to PTVs or to radiation doses, such as coverage of the contralateral neck	small changes to PTVs and contours, such as minor extension or reduction of coverage
Huo	2022	R	Canada	SBRT	Weekly	N	Targets, OARs	Critical; non-critical	Errors requiring immediate plan changes due to potential compromise of patient safety/treatment quality	Non-urgent and non-critical error types
Khader	2019	R	Jordan	All	2-3 per week	N	NR	Major; minor	Any substantial change which requires patient re-simulation and/or re-planning.	Any plan change which doesn’t necessarily require re-planning.
Lymberiou	2015	P	Canada	BR	Weekly	N	All	Grades A, B, C	Unsatisfactory and require correction before the next	Satisfactory to continue treatment but receive

© 2024 Hughes RT et al. *JAMA Network Open*.

									fraction of radiation therapy is delivered	suggestions for potential changes that should be incorporated into similar plans in the future
Mackenzie	2016	P	Canada	HN, LU, BR	Weekly	N	Targets, OARs	Grades A, B, C	Unsatisfactory and require correction before the next fraction of radiation therapy is delivered	Satisfactory to continue treatment but receive suggestions for potential changes that should be incorporated into similar plans in the future
Martin-Garcia	2020	P	Spain	All	Daily	N	Targets, OARs	Major; minor	NR	NR
Matuszak	2016	R	US	SBRT	Daily	N	GTV, PTV	None	NA	NA
McClelland	2021	R	US	All	Daily	N	Targets, OARs	Major; minor	Any suggested plan revision strongly recommended before administration of the next fraction of treatment	Any suggested plan revision not required for treatment
Mitchell	2017	R	US	All	3-4 days weekly	N	Targets, OARs	Major deviation; variation	I would manage this case differently. The current plan is	I would manage this case differently, but

© 2024 Hughes RT et al. *JAMA Network Open*.

									not reasonable. I recommend changes be made.	the current management plan is reasonable
Qureshi	2019	P	Pakistan	All	Weekly	N	Targets	Major; minor; missing contour	Treatment volumes, fields, or doses not acceptable	Changes made within treatment volumes, fields
Ramasamy	2019	R	UK	HN	Weekly	N	GTV, CTV	Major; minor	Alteration to the GTV and/or high-dose CTV and/or the prescribed dose or fractionation	Alteration to the intermediate- or elective-dose CTV
Rooney	2015	P	UK	LU	Weekly	N	Targets, OARs	None	NA	NA
Samuel	2019	R	UK	HEME	Weekly	N	GTV, CTV	None	NA	NA
Tchelebi	2023	P	US	All	Daily	N	All	Grades A, B, C	Major deficiencies such as grossly incorrect or incomplete contours, significant required modifications to the prescription or written directive, laterality modifications, or inappropriate utilization of RTT	Minor modifications such as small modifications to the target volumes or organs at risk, or if there were minor errors or discrepancies in the written directive and prescription
Zairis	2017	P	US	HN	Weekly	N	All	Major; minor	NR	NR

© 2024 Hughes RT et al. *JAMA Network Open*.

eTable 2. Intent of Treatment

Study	Year	Total Number	Total Curative	Definitive	Adjuvant or Neoadjuvant	Palliative
Adjogatse	2023	50	48	37	11	2
Ali	2024	1160	NR	NR	NR	NR
Ballo	2014	2830	NR	NR	NR	NR
Bhattacharyya	2024	204	204	151	53	0
Brammer	2014	152	NR	NR	NR	NR
Braunstein	2017	80	80	38	42	0
Cardenas	2016	85	85	37	48	0
Chiu	2021	73	73	50	23	0
Cooper	2020	222	NR	NR	NR	NR
Dimigen	2014	99	NR	NR	NR	NR
Farris	2023	35	35	35	0	0
Farris	2022	208	194	91	103	11
Fitzgerald	2020	169	NR	NA	NA	NA
Fong	2017	62	62	43	22	0
Gatfield	2023	120 (53:67 Y/N)	NR	NR	NR	NR
Gogineni	2023	360	360	193	167	NR
Hesse	2022	511	NR	NR	NR	NR
Huo	2022	934	NR	NR	NR	NR
Khader	2019	7149	NR	NR	NR	NR
Lymberiou	2015	2223	2223	NR	NR	0
Mackenzie	2016	257	239	NR	NR	18
Martin-Garcia	2020	148	122	65	57	19
Matuszak	2016	513	NR	NR	NR	NR
McClelland	2021	469	327	NR	NR	142
Mitchell	2017	442	NR	NR	NR	NR
Qureshi	2019	116	96	NR	NR	20
Ramasamy	2019	307	307	195	112	NA
Rooney	2015	122	122	NR	NR	0
Samuel	2019	158	NR	NR	NR	0
Tchelebi	2023	20069	15659	NR	NR	4410
Zairis	2017	182	NR	NR	NR	NR

© 2024 Hughes RT et al. *JAMA Network Open*.

eTable 3. Quality of Included Studies Assessed by the MINORS Criteria

Study	Year	Clearly stated aim	Inclusion of consecutive patients	Prospective data collection	Endpoints appropriate of study	Unbiased assessment of study endpoint	Total score
Adjogatse	2023	2	2	2	2	1	9
Ali	2024	2	2	0	1	2	7
Ballo	2014	2	2	0	2	2	8
Bhattacharyya	2024	2	2	2	2	1	9
Brammer	2014	2	0	1	1	1	5
Braunstein	2017	2	2	0	2	2	8
Cardenas	2016	2	2	2	2	2	10
Chiu	2021	2	2	1	2	2	9
Cooper	2020	2	2	2	1	1	8
Dimigen	2014	2	1	0	1	1	5
Farris	2022	2	2	2	2	2	10
Farris	2023	2	2	2	2	2	10
Fitzgerald	2020	2	1	2	1	1	7
Fong	2017	2	2	2	2	2	10
Gatfield	2023	2	2	2	2	2	10
Gogineni	2023	2	2	2	2	2	10
Hesse	2022	2	1	0	1	2	6
Huo	2022	2	1	1	1	1	6
Khader	2019	2	1	1	1	2	7
Lymberiou	2015	2	1	1	2	2	8
Mackenzie	2016	2	1	1	2	2	8
Martin-Garcia	2020	2	1	1	1	1	6
Matuszak	2016	2	1	1	1	2	7
McClelland	2021	2	1	0	2	1	6
Mitchell	2017	2	1	1	2	2	8
Qureshi	2019	2	2	2	2	2	10
Ramasamy	2019	2	1	1	2	2	8
Rooney	2015	2	2	2	1	1	8
Samuel	2019	2	1	1	2	2	8
Tchelebi	2023	2	1	1	2	2	8
Zairis	2017	2	2	2	2	2	10

© 2024 Hughes RT et al. *JAMA Network Open*.
